# Patients' Voices of Care Encounters and Organisational Influences on Existential Suffering in Cancer Care: A Qualitative Study

**DOI:** 10.1111/scs.70293

**Published:** 2026-07-02

**Authors:** Cecilia Linnanen, Elisabeth Bergdahl, Jessica Hemberg

**Affiliations:** ^1^ Department of Health Sciences, Faculty of Science and Engineering Åbo Akademi University Vaasa Finland; ^2^ School of Health Sciences Örebro University Örebro Sweden

**Keywords:** caring, encounter, existential suffering, healthcare management, suffering, unmet care needs

## Abstract

**Introduction:**

Receiving a cancer diagnosis is often experienced as a violent life turn, evoking existential suffering. To promote health and alleviate suffering, it is crucial to acknowledge and respond to patients' existential needs. However, nurses frequently lack the time and resources to address these needs, and when cancer care is reduced to merely treating the physical body, it may increase suffering and neglect the person as a whole.

**Aim:**

The aim of this study was to gain a deeper understanding of patients' existential suffering and what they need to alleviate suffering in cancer care.

**Methods:**

A qualitative inductive design was used. The data consisted of in‐depth interviews with 10 Finnish women with cancer or cancer survivors. A qualitative content analysis was conducted.

**Findings:**

The study found three themes: (1) Inner experiences and need for human connection; (2) Encounters with healthcare professionals can either cause or alleviate patients' suffering; and (3) The organisation's impact on patient suffering.

**Conclusions:**

This study demonstrates that existential suffering in cancer care is shaped not only by the illness itself but also by care encounters and organisational conditions. Fragmented care and lack of continuity may intensify suffering, whereas compassionate, person‐centred encounters can alleviate it. Addressing these issues may reduce patient suffering, enhance patient safety, support healthcare professionals, and contribute to a more sustainable healthcare system.

## 1 Introduction

Cancer occurs worldwide, causing close to ten million deaths globally every year, and the most common forms are breast, lung, colon and prostate cancers [1]. In Finland, cancer is the second most common cause of death [2]. However, cancer is more than just a medical diagnosis; it is deeply personal, with unique human stories of suffering, grief, healing, resilience and love [3]. Nurses provide up to 80% of patient care [4], but they often feel that they lack the time or resources to address patients' psychosocial and existential needs [5]. Cancer care reduced to only the treatment of the body can cause suffering and unmet patient needs [6]. Cocreated and person‐centred care [7], on the other hand, can address these issues by holistically meeting the patient's individual needs, thereby improving the quality of care. Existential aspects of suffering remain insufficiently addressed in cancer care and research, leaving a gap in understanding existential needs in clinical practice [8, 9]. This study aims to address this gap by providing an in‐depth exploration of patients' perspectives.

## 2 Background

Receiving a cancer diagnosis is generally experienced as a violent life turn described as existential suffering in the form of fear, powerlessness, frustration, loss, grief, pain, anxiety, loneliness, and a confrontation with death [10, 11, 12, 13]. Existential suffering is described as shame, worthlessness, hopelessness, meaninglessness, fear of death, despair, abandonment, bodily discomfort, pain, and a desire to die [9, 14, 15]. Physical pain is said to be easier to bear than existential pain [6]. Cancer patients experience increased dependence on others and struggle with loss of independence, challenging their dignity, integrity, and identity, and leading to a fear of being a burden [13]. Life after cancer is described as unfamiliar fatigue, limited life space, ongoing inner struggle, and bearing the burden alone [16, 17].

Existential needs, such as finding hope, discussing death, and sharing thoughts and feelings with loved ones, are common among people with cancer, and close human contact is crucial for finding a reason to live [11, 18]. To promote health and alleviate suffering, care must be grounded in approaches that communicate recognition, dignity, and value to patients [19, 20]. Continuity is crucial, meaning that patients have the same caregiver who knows them well, fostering trust [21]. Social support and hope positively impact resilience, whereas mental and physical burdens negatively affect it [8, 22].

If the existential dimension of the human being is not acknowledged, there is a risk of dehumanising care situations and overly objectifying the patient, causing more suffering and increasing the likelihood of unmet care needs and needing more care in the future [6, 9, 12, 23]. In healthcare, suffering can be caused by disrespect or depriving the patient of dignity [19]. Ignoring existential anxiety early in cancer care can lead to isolation, introversion, and reduced help‐seeking due to a lack of trust in healthcare professionals during times of great distress [14, 24].

Organisational fragmentation, task‐oriented care models, and efficiency‐driven routines have been shown to compromise human connection and individual care needs in cancer care, contributing to increased suffering [23, 25, 26]. Cancer care in Finland is focused on clinical care, and much needed psycho‐social‐existential support is lacking [27]. In this study, psychological difficulties are understood as emotional and cognitive responses to illness, whereas existential suffering refers to a deeper disturbance related to meaning, dignity, and the experience of being human in the face of illness and mortality [9, 28]. Relatively few qualitative studies have focused on the suffering of cancer patients, which creates a knowledge gap that makes it relevant and important to explore.

## 3 Aim

The aim of this study was to gain a deeper understanding of patients' existential suffering and what they need to alleviate suffering in cancer care.

## 4 Theoretical Perspective

This study's theoretical perspective is based on Katie Eriksson's theory of human health, suffering, and caritative caring. Health is seen as alleviated suffering and an inseparable whole of the body, mind, and spirit [29]. Suffering is unique to each individual, and recognising it provides comfort, while ignoring it can lead to feelings of abandonment and despair [28]. Alleviating suffering involves feeling loved, acknowledged, and understood and receiving necessary care [28]. Caritative caring emphasises compassionate relationships, aiming to alleviate suffering, promote health and protect life through genuine caring and a willingness to help [30]. Eriksson's theory was chosen during the planning phase of the study because it offers a comprehensive understanding of suffering, which aligns with the study's aim. The theory served as a pre‐understanding that shaped the research aim, approach, and the interview guide.

## 5 Methods

A qualitative inductive design was employed [31] to generate findings through an active analysis of participants' accounts, grounded in their own words, in an area with limited prior research. The study was reported in accordance with the COREQ (Consolidated Criteria for Reporting Qualitative Research) checklist.

### 5.1 Participants and Recruitment

Ten Finnish women aged 32–72 years participated in the study. All had current or previous experience of cancer (brain, intestinal, blood, breast, or ovarian) and were in different phases of the cancer care trajectory, including curative, palliative, and survivorship phases. Participants were recruited from different regions of Finland and had illness experiences ranging from recently initiated treatment to living with cancer over several years.

Participants were recruited using purposive, self‐selected sampling via a Finnish cancer society, where the study invitation was disseminated face‐to‐face by nurses as well as through the organisation's online platforms. Nine participants were recruited in this way, and one additional participant was recruited through an open Facebook post. Adult individuals with personal experience of cancer‐related suffering were invited to participate through a study invitation targeting experiences of severe suffering or degrading treatment in healthcare. Interested individuals contacted the first author by email or telephone, received information about the study, and an interview was scheduled. All individuals who expressed interest met the inclusion criterion and were included. No participants declined participation or withdrew after informed consent had been given.

### 5.2 Data Collection

Semi‐structured individual interviews were conducted in Finnish by the first author during the winter of 2022–2023. Prior to each interview, the interviewing researcher introduced herself and provided information about the study. An interview guide was specifically developed for the present study, supported by Eriksson's caritative caring theory. The guide was not pilot tested but was used with follow‐up questions to deepen and clarify participants' narratives. Field notes were taken during and after the interviews. Participants were invited to speak freely about their cancer illness, their experiences of suffering related to cancer and care encounters, and what alleviated suffering. Interview questions were not provided in advance, in order to support open and spontaneous narratives.

The interviews were conducted via video call or telephone with at‐home participants and lasted between 60 and 150 min. Only the participants and the first author were present during the interviews. All interviews were digitally recorded and transcribed verbatim for analysis. After ten interviews, data saturation was discussed and confirmed by two researchers.

### 5.3 Data Analysis

A qualitative content analysis was conducted according to Lundman and Hällgren Graneheim [31]. The study aim guided the entire analysis. The first and last researchers read the data multiple times to gain an overall understanding. Meaning units were identified, condensed, and coded by the first author, using Microsoft Excel to manage the data. These codes were continuously compared and grouped into subcategories and categories, which were subsequently abstracted into themes reflecting the underlying meanings in the data.

Although Eriksson's theory informed the researchers' pre‐understanding, it was set aside during the analysis to remain open to new insights. No themes were identified in advance, and the analysis was inductive and data‐driven. The analytic process was iterative, involving movement between the data and successive levels of interpretation [31]. A synthesis was developed in the form of a model. Consistency between the data and findings was discussed and confirmed by the first and last authors. Credibility was enhanced through researcher triangulation and the use of illustrative quotations. For an example of the analysis, see Table 1.

**TABLE 1:** An example of a completed analysis.

Meaning unit	Code	Subcategory	Category	Theme
‘*It was perhaps the worst moment in the hospital. That … I was so terribly helpless and in distress with the terrible pain. And then when at last I finally asked for help, […] they did not listen but walked all over me*’ (P10)	It was very hurtful, that they didn't take my pain seriously and didn't listen	To be left to one's fate, abandoned	The encounter with healthcare professionals can cause the patient's suffering	Encounters with healthcare professionals can either cause or alleviate the patients' suffering
‘*When someone listens, understands, and says that you have the right to feel this way, and that way, and that way. […] that was something that made me feel like maybe I can get through this*’ (P8)	When someone listens and understands	To feel that someone truly cares	The humane encounter with healthcare professionals alleviates patient suffering	

### 5.4 Ethical Considerations

This study adhered to the principles of responsible and ethical conduct of research with human participants, aiming to avoid exploiting, harming, or hurting any individual, in line with the guidelines of the Finnish National Board on Research Integrity [32, 33]. Ethical permission was granted by the research ethics board of the university of the first and last researchers and the chosen cancer society (FEN ÅA 30/5/2022). Participation was voluntary. Participants received comprehensive information about the study prior to the interviews, and written and verbal consent was obtained. The participants' identities remained confidential throughout the study, and all the data were stored on two local computers secured with locks and passwords.

## 6 Findings

**FIGURE 1:**
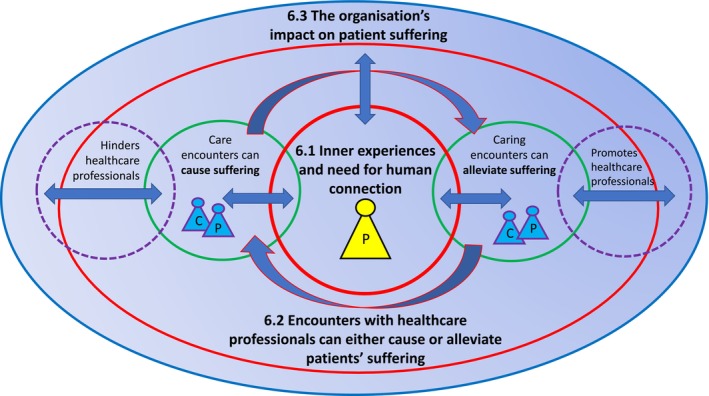
Overview of the findings. C = healthcare professional, P = Patient.

The individual's existential suffering in cancer care is influenced by encounters with healthcare professionals. Encounters with healthcare professionals take place within an organisational context that affects patients' existential suffering both directly, through experiences of fragmentation and unmet needs, and indirectly by promoting or hindering healthcare professionals' capacity to engage in caring encounters that may alleviate suffering or contribute to encounters that cause suffering. The analysis generated three themes and five categories. Figure 1 illustrates how the themes are interrelated in the model, while the text provides detailed descriptions of the categories.

### 6.1 Inner Experiences and Need for Human Connection

#### 6.1.1 The Inner Experience

Based on the participants' statements, cancer and its treatment can cause strong and difficult feelings. The participants described their inner experiences as including self‐hatred, feeling like a burden, guilt, vulnerability, distress, sadness, loneliness, disappointment, loss, injustice, helplessness, hopelessness, fear, darkness, insecurity, paralysis, lack of control and concern for loved ones. One participant mentioned thirsting for information and spiritual strength to keep going. Another participant said:

I was in a terrible condition during that cytostatic care; I couldn't move anywhere. I was exhausted. […] Nothing was a given. When you took 1 min at a time, the suffering was there, completely present. (P9)

Throughout their illness, all participants wrestled with the inner existential threat to life itself, as death emerged not merely as a distant possibility, but as a more concrete presence. One participant said: ‘*I got such symptoms that I didn't want to live. […] I kind of exploded inside, and then my heart started beating like crazy, and it was absolutely terrible!*’ (P2). Most recounted that during severe suffering, the idea of death seemed like a relief.

The participants identified various factors as the most difficult aspects of suffering. For some, it was physical pain; for others, spiritual anguish; for several, it was disrespectful encounters with healthcare professionals; and for others, it was the loss of health and joy in life. One participant expressed, ‘*I truly lost my whole life, that is, my whole life, and it has been very hard*’ (P5). Most participants felt that being a burden to others caused severe suffering. Many were concerned about how their family would cope with their death. Some wished to die to stop being a burden or to avoid struggling with healthcare professionals.

The participants also faced other burdens and losses, such as loss of work, travel, or pets, which added to their suffering. For example, the COVID‐19 pandemic and subsequent restrictions caused loneliness when support from relatives was most needed. Some participants lost their ability to walk during cytostatic treatment, and this ability did not return after treatment, causing great loss and disappointment.

#### 6.1.2 Need for Human Connection

The participants noted that suffering had different stages over time. There was a state of acute suffering, where individuals were just trying to survive moment to moment. One participant described it as a ‘fighting will’. For some, the diagnosis and ensuing shock and fear of death triggered this survival state, while for others, it was pain or severe nausea. One participant said:

It was somehow really important also in accepting the illness, or in the early stages, when you saw your close loved ones and were able to talk to them, see them, and touch them. That was incredibly important. (P8)

The participants described close human contact as extremely important when suffering was at its worst. Many participants in the survival state were not ready to talk about deeper feelings, using all their energy to get through each moment. They wanted to forget. Simple conversations, such as discussing the weather or family, helped distract one from acute suffering.

After acute suffering, a processing state emerged that also required care. For some, this came earlier; for others, it came a longer time after cancer care ended. One participant said:

I have experienced that shortly after the acute phase is over, they forget about the human being. It would be very important at the time, when you're processing things, to allow time for those questions. (P10)

In this state, the participants wanted to sort out events and difficult emotions. Talking to someone helped the most, whether a psychologist or healthcare professional who could answer specific questions about their cancer care journey. Many found it difficult to access cancer care professionals during the processing state.

### 6.2 Encounters With Healthcare Professionals Can Either Cause or Alleviate Patients' Suffering

#### 6.2.1 The Encounter With Healthcare Professionals Can Cause Patient Suffering

All the participants recounted difficult experiences with healthcare professionals (both nurses and physicians). For several, disrespectful encounters caused the worst suffering, even more than severe physical pain. The participants described suffering as belittling, neglecting, lacking trust, arrogance, illogical, degrading, abusive, tormenting, superiority, and not listening, along with inner disappointment, helplessness, and feelings of abandonment. Some felt threatened by healthcare professionals. One said:

And I said, ‘Don't take it [the epidural] away’. But no, and you know, they just swung me on my side and removed the epidural, and after that all hell broke loose. And for 24 h, the pain was such that I could not lift a finger, so hard was the pain. It was a terribly traumatic experience! […] And I just told [my husband] ‘Can you get me out of here? I don't want to be here anymore, I dare not be here anymore!’ (P6)

Most participants lost their trust in healthcare professionals when their needs were not listened to or understood, feeling abandoned and stripped of dignity. One described it as feeling raped. Several found it difficult to depend on others' grace. One participant reported:

I almost thought of these German human experiments during the war, all the gas chambers and these, I felt I was a laboratory animal who has no power at all. (P1)

The participants felt that they were just bodies needing care and were not seen as people beyond their physical illness. Nurses did not discuss the cancer or its emotional impact, nor did they inform patients about different forms of support. One participant reported:

It was perhaps the worst moment in the hospital. That … [crying] I was so terribly helpless and in distress with the terrible pain. And then when I tried to the last moment and a little more to cope with the pain, […] thinking all the time that it will pass, and then when at last I finally asked for help, and I was in terrible distress, they did not listen, but walked all over me. (P10)

The participants repeatedly brought up that they had to struggle to have their needs met, seeking support alone, for example, social services, taxi subsidies, and psychological or peer support, which was burdensome. Feeling abandoned when in great need of support caused feelings of unsafety. The participants recounted needing to be on guard during care due to professionals forgetting allergies, pain history, or special needs, fearing care errors.

#### 6.2.2 The Humane Encounter With Healthcare Professionals Alleviates Patient Suffering

The participants felt that humane encounters during severe suffering was crucial. Being seen as a whole person, not just a task, was alleviating. They wanted healthcare professionals to listen, believe in them, and show compassion and understanding. That someone truly cared. Getting to know the person behind the patient was important. Some described how a nurse's soft demeanour and step‐by‐step explanations during an examination made them feel safe in a frightening situation. One participant said, ‘*[The feeling of dignity] was infinitely important*’ (P6). Being cared for in this way led to a feeling of trust and security. Even small gestures made the participants feel seen. One participant described:

When someone listens, understands, and says that you have the right to feel this way, and that way, and that way. […] that was something that made me feel like maybe I can get through this. (P8)

Allowing emotions to come out was liberating and relieving, as the whole self was accepted. Another participant stated:

Sometimes during the night shift, there were some wonderful nurses who would stay and sit with me if things weren't too busy. We would talk about my studies […]. Those who had the patience to stay and sit with me when I couldn't sleep because of the pain, or couldn't rest because the neighbour was snoring, or something else—there were those [nurses] who wanted to connect, talk, and be present. (P4)

The participants wanted nurses to have time to talk, be present and look them in the eye. Understanding special situations, such as offering a sex therapist after a mastectomy or noting important conditions in the patient record, was important. The participants highlighted the need to talk about difficult experiences, whether with a psychologist or the professionals who treated them. One participant had a need to discuss death openly without being rejected.

### 6.3 The Organisation's Impact on the Patient Suffering

#### 6.3.1 Experiences of Fragmentation and Unmet Needs

The healthcare organisation has a direct impact on patient suffering. The participants conveyed that cancer care is very fragmented and described it as being put on a train, moving from one care situation to another. One participant stated, ‘*I experienced that here you only take care of this operation and that's it. And then you are transferred. There was no specific discussion…*’ (P4). One hospital handled tumour operations, another provided cytostatic treatment, and a third offered radiation therapy. Participants felt that communication between different instances was lacking. Patient counselling was outsourced to psychiatry. Most participants felt that they lacked information and were lost when navigating the various support options they needed. The participants expressed a desire for more holistic care, including conversational therapy as a natural part of cancer care, even after physical treatments ended.

The participants expressed that the nurses were always in a hurry and too tired to talk to patients. One participant noticed a power imbalance between nurses and physicians, causing suffering when nurses noticed something wrong but ‘*could not defy authority*’ (P2). The participants felt that giving feedback would not change anything, or feared being labelled difficult and not receiving future care. Obtaining financial compensation for taxi subsidies was challenging due to bureaucracy and regulations, leaving patients unsupported. One participant related:

From the cancer outpatient clinic, they wrote one of those treatment letters that they will not take a position on my pain medication at all, because according to them this belongs to the health station, and according to the health station, of course it belongs to the cancer outpatient clinic, because it is the cancer that causes this pain. (P5)

Participants felt thrown out of the care system when their needs did not fit predetermined ‘boxes’. One participant said, ‘*When you have to go time and time again, and you are in no way heard or seen regarding the matter*’ (P9). Healthcare was seen as rigid and system‐focused, not allowing room for the individual needs of the person as a whole.

## 7 Discussion

The aim of this study was to gain a deeper understanding of patients' existential suffering and what they need to alleviate suffering in cancer care. The findings show that both cancer and its care can give rise to existential suffering, manifested as difficult emotions, vulnerability, distress, and loss, which is consistent with previous research [6, 10, 12]. Participants described losing important aspects of life due to cancer, such as the ability to walk, work, or travel [14, 30], which in turn contributed to suffering related to feeling like a burden to others [13]. At the same time, it is important to acknowledge that not all patients experience extensive psychological needs in relation to cancer care, as noted by Furmenti et al. [34].

Participants' experiences of abandonment and suffering due to compromised dignity are consistent with previous studies [19, 35, 36] and can be further interpreted through Eriksson's [30] understanding of suffering from care as a violation of human dignity. While previous research has shown that care encounters may contribute profoundly to patients' suffering [6], the present study adds that suffering caused by care may further aggravate the suffering related to the illness. This finding emphasises that existential suffering is not limited to inadequate or erroneous care but may also arise within technically correct and guideline‐based cancer care.

Previous studies have often conceptualised existential suffering in cancer as a state or condition [12, 13, 15]. While these studies provide important insights into the nature of existential suffering, the present study contributes an increased understanding of how suffering evolves over time, shifting between acute survival‐oriented states and later phases of processing. Although Ellingson and Borofka [37] and Ueland et al. [16] approach existential suffering as a lived experience over time, the present study extends this knowledge by demonstrating how the process of existential suffering is impacted by care encounters and organisational structures. The evolving nature of suffering can be interpreted through Eriksson's [28] drama of suffering, in which being in suffering may unfold into reconciliation through the caring relationship. This understanding resonates with Morse and Carter's [38] conceptualisation of enduring and emotional suffering as dynamic processes evolving toward acceptance and the reformulation of the self.

According to the participants, some healthcare professionals were able to alleviate suffering through good encounters, despite time constraints. These findings raise questions as to whether certain professionals may adopt more caring approaches, or develop forms of resistance to the negative impacts of contemporary care work, a phenomenon that warrants further investigation. This underscores the importance of empathetic care grounded in listening, dignity, and openness to coming to know patients as whole human beings over time [19, 35, 36, 39]. Across the narratives, participants expressed a clear wish for mental well‐being to be acknowledged and supported throughout the care trajectory. The integration of other caring professionals, such as psychosocial, existential, or spiritual care professionals, may further support patients' existential needs and alleviate suffering as part of an interprofessional approach to cancer care [40, 41, 42]. Nevertheless, most participants described their care as lacking the person‐centred and co‐created approach outlined by Hemberg and Bergdahl [7], despite expressing a clear preference for such care.

The findings further indicate that patients suffer within a care culture that prioritises the physical body and disease over the person as a whole [5, 9, 12, 35]. Walley et al. [43] describe how such a focus may lead to failure demand, as the healthcare system fails to detect patient needs, resulting in repeated patient contacts and longer queues. However, patients may also refrain from seeking care due to damaged trust [24]. Somatic cancer care may thus contribute to severe existential suffering, leading to future psychiatric care needs that could be avoided through timely and appropriate support [44]. Organisational constraints prevent healthcare professionals from forming continuous relationships with patients, often resulting in frustration [42]. In parallel, nurses are themselves affected by patient suffering and want to care; however, repeated exposure to demanding situations combined with limited time may lead to compassion fatigue [45].

## 8 Methodological Considerations

An inductive, qualitative approach with in‐depth interviews is considered suitable for interpreting and understanding existential suffering and its alleviation [46, 47]. The sample was diverse, representing participants from different regions of Finland, a range of cancer diagnoses, and varied ages. Descriptive quotations enhance the credibility of the findings, and the methodological and analytical processes are thoroughly documented to ensure transparency [47]. The first and last researchers discussed the analysis in detail, and all researchers contributed to the final themes [48]. The sample size was guided by data saturation rather than numerical representativeness, as the interviews yielded rich and comprehensive data to support the development of categories and themes [49].

One limitation of this study is that it included only female participants. However, a strength is the rich data it yields. The study's broad range of patient groups, including cancer survivors and patients in the curative and palliative stages, may be seen as a weakness. The recruitment method likely yielded a group with severe suffering related to care encounters. These findings cannot be generalised to all cancer patients but provide deeper insight into the existential suffering that may occur in cancer care [46]. Quantitative studies are needed to assess the prevalence of extensive existential suffering among cancer patients.

The participants expressed that the study addressed important issues for improving healthcare, enhancing the study's credibility. Having their voices and experiences heard was a source of relief for the participants. One participant reviewed and confirmed the findings [46], while the interview transcripts were not returned to participants for review. The researchers had an open attitude and felt a strong responsibility to present the experiences as described by the participants. The researchers did not know the participants beforehand.

## 9 Conclusions

This study provides new insights into existential suffering by describing its processual nature, illustrating how patients move between acute suffering and later phases of processing. A key contribution of this study is that suffering is not only related to the illness itself but is also impacted by care encounters and organisational conditions. Importantly, the findings show that suffering arising from care may aggravate the illness‐related suffering, and may occur even within technically correct cancer care.

Fragmented care and lack of continuity were found to increase existential suffering, whereas compassionate, person‐centred encounters and human connection alleviated suffering and fostered trust. These findings highlight the need to integrate existential support with somatic cancer care and to develop flexible organisational models that enable caring relationships.

The findings are relevant for healthcare leaders, cancer care professionals, and educators, highlighting ethical responsibilities to recognise and respond to existential suffering as an integral part of organisational structures and quality of care to reduce patient suffering. Future research should explore organisational and professional conditions that enable or hinder caring encounters, and examine unmet care needs and failure demand, for example through an action research approach involving health care professionals in bottom‐up, practice‐based inquiry and development. Addressing these issues has the potential to alleviate patient suffering, improve patient safety, support healthcare professionals, and contribute to a more sustainable healthcare system.

## Author Contributions

Study design: Cecilia Linnanen and Jessica Hemberg. Data collection: Cecilia Linnanen. Data analysis: Cecilia Linnanen and Jessica Hemberg. Study supervision: Elisabeth Bergdahl and Jessica Hemberg. Manuscript writing: Cecilia Linnanen, Elisabeth Bergdahl and Jessica Hemberg. Model development: Elisabeth Bergdahl. Critical revisions for important intellectual content: Cecilia Linnanen, Elisabeth Bergdahl and Jessica Hemberg.

## Funding

This work was supported by the Svensk‐Österbottniska Samfundet (9498/10270), the Åbo Akademi University Foundation (2802110), the Åbo Akademi, Solutions for Health (28200325K22), the Swedish Cultural Fund (192915), the Ethel Tybeck Fund (226646).

## Disclosure

Software: Express Scribe was used to support the transcription process. Microsoft Excel was used to support data management and analysis. A locally hosted version of M365 Copilot, accessible only via secure university login and not connected to the public internet, was used solely to assist with language editing and proofreading of the manuscript.

## Consent

Participation was voluntary during the whole study. Written and verbal informed consent was obtained from all participants before the study. Participants received both written and oral information about the study before the interviews.

## Conflicts of Interest

The authors declare no conflicts of interest.

## Data Availability

Owing to the sensitive nature of the interview data involving patients suffering from cancer, the transcription data are not publicly available. It is kept confidential by the corresponding author to protect the privacy and well‐being of the participants.
